# Mesenchymal Stem Cell Derived Exosomes Alleviates Hirschsprung-Associated Enterocolitis by Inhibiting AKT Phosphorylation in Macrophages Through miR-223

**DOI:** 10.1155/sci/3218993

**Published:** 2025-09-15

**Authors:** Haosen Ji, Zheming Xu, Leiting Shen, Sisi Yang, Jingyi Jin, Chengjie Lyu, Yichao Ren, Yi Xiao, Yuebai Zhang, Shu Fang, Xiaoxia Zhao, Xiang Yan, Dengming Lai, Jinfa Tou

**Affiliations:** ^1^Department of Neonatal Surgery, Children's Hospital, Zhejiang University School of Medicine, National Clinical Research Center for Child Health, Hangzhou, China; ^2^Department of Urology Surgery, Children's Hospital, Zhejiang University School of Medicine, National Clinical Research Center for Child Health, Hangzhou, China; ^3^Department of Thoracic and Cardiovascular Surgery, Children's Hospital, Zhejiang University School of Medicine, National Clinical Research Center for Child Health, Hangzhou, China

**Keywords:** exosome, Hirschsprung-associated enterocolitis, macrophages, mesenchymal stem cells, miR-223

## Abstract

**Background:** Mesenchymal stem cells (MSCs) inhibit macrophage inflammatory response and alleviate intestinal inflammation. However, the role of MSCs in Hirschsprung-associated enterocolitis (HAEC) remains uncertain. This study aims to investigate the effects of MSCs on HAEC and the mechanisms related to macrophages and MSCs.

**Methods:** Immunofluorescence was used to measure CD68 and p-AKT in colonic tissues of HSCR patients with HAEC. Ednrb^−/−^ mice was used as HSCR model. The proportion of colonic tissue macrophages in WT and Ednrb^−/−^ mice was assessed by flow cytometry. The colonic tissues injury was evaluated with HE staining and the survival curves of mice were recorded. In vitro, macrophage-induced enterocyte death was induced by lipopolysaccharide (LPS). MSCs, MSC derived exosomes, miR-223, or MK2206 were added to macrophages, and the levels of miR-223 in macrophages after exosome treatment were measured by RT-qPCR. Flow cytometry was used to assess enterocyte death, western blot was performed to measure p-AKT expression in macrophages, and enzyme-linked immunosorbent assay (ELISA) was used to detect IL-1β concentration in macrophage supernatants and serum of Edrnb^*−/−*^ mice.

**Results:** Increased expression of CD68 and p-AKT was observed in the colonic tissues of HAEC patients. Colonic instillation of MSCs derived exosomes significantly reduce the inflammatory score of colonic tissues and prolong the survival time of HAEC mice. In vitro, LPS-stimulated macrophages induce the phosphorylation of AKT and enterocyte death. Stimulation of macrophages with MSC-derived exosomes increased the content of miR-223. MSC-derived exosomes, miR-223 and MK2206 significantly reduce macrophage-induced enterocyte death, attenuated AKT phosphorylation in macrophages, and decreased IL-1β concentration in macrophage supernatants.

**Conclusion:** Macrophages accumulate in colonic tissues during HAEC and inflammatory macrophages drive enterocyte death. MSCs derived exosomes reduce enterocyte death by suppressing AKT phosphorylation and IL-1β secretion via miR-223, and subsequently mitigate HAEC in mice. These findings suggest that MSC-derived exosomes, particularly those enriched in miR-223, may serve as a promising therapeutic strategy for the prevention or treatment of HAEC.

## 1. Introduction

Hirschsprung's disease (HSCR) is a common congenital intestinal disease with an incidence of approximately one in 5000 live newborns [1]. Its main pathological feature is the absence of ganglion cells in the myenteric and submucosal neuron of the colon. Hirschsprung-associated enterocolitis (HAEC) is a common complication of HSCR. HAEC occurs before and after surgical removal of the diseased colon, and recurrent episodes significantly impact the quality of life and even pose life-threatening risks to affected individuals [2–4]. Currently, the exact mechanism underlying HAEC remains unclear.

Various risk factors influence the occurrence of HAEC in clinical practice, including gender, age at diagnosis, family history, time of diagnosis, surgical procedure, and the length of the colon segment affected by HSCR [5]. The precise cause of HAEC is not yet fully understood, but recent research suggests that it may involve multiple factors such as genetic inheritance, gut microbiota, intestinal barrier function, enteric nervous system, and immune system [6]. Further exploration of the pathogenesis of HAEC can provide new directions for its prevention and treatment [7, 8]. Experimental evidence has shown significant changes in gut bacterial abundance and composition before the onset of HAEC in mouse models [9]. Alongside dysbiosis, there is a notable decrease in secretory phospholipase A2 levels, leading to compromised mucosal barrier function [10]. The increased proportion of abnormal microbial species elevates the levels of lipopolysaccharide (LPS) in the colon, which significantly increases intestinal epithelial permeability. LPS, along with damage-associated molecular patterns released by damaged epithelial cells, activates the toll-like receptor (TLR) in macrophages, stimulating the NF-κB signaling pathway and inducing increased expression of inducible nitric oxide synthase [11]. As a result, macrophages polarize towards an M1 phenotype, releasing a large amount of inflammatory cytokines such as tumor necrosis factor-alpha (TNF-α), interleukin-1β (IL-1β), nitric oxide, and chemokines involved in lymphocyte and monocyte recruitment (CCL-17 and CCL-24) [11]. Among these, TNF-α and IL-1β induce the expression of E and P selectins on endothelial cells, leading to the influx of circulating monocytes and lymphocytes into the damaged intestine. CCL-17 and CCL-24 recruit M1 macrophages and CD4^+^ Th1 cells, exacerbating colonic inflammation-induced injury [12]. M1 macrophages also damage Cajal cells, resulting in impaired intestinal motility, further worsening intestinal inflammation and creating a vicious cycle [13–16]. Previous studies have identified macrophages as crucial immune cells in colonic inflammation and emerging targets for immunotherapy of intestinal inflammatory diseases [12, 17, 18].

Mesenchymal Stem Cell (MSC) is a type of multipotent adult stem cell derived from the mesoderm. It owns self-renewal capacity and can differentiate into various cell types. Due to its low tumorigenicity and low immunogenicity, MSC has found broad applications [19–21]. Previous research on HSCR and related diseases has primarily focused on stem cell differentiation and intestinal function restoration, overlooking the immunomodulatory effects. MSCs regulate the function of innate immune cells through their exosomes [22], exerting immunosuppressive effects to alleviate intestinal inflammation. The main component responsible for the effects of MSC-derived exosomes is miRNA [23]. By inhibiting mRNA and regulating the expression of target genes, miRNA participates in various biological processes [24]. Although studies have confirmed the role of miRNA in MSC-derived exosomes in modulating macrophage function and alleviating various intestinal inflammations [22, 23, 25], their specific role and mechanisms in HAEC remain unclear.

A recent study has identified the PI3K/AKT signaling pathway as an important regulator of intestinal inflammation [26]. AKT, also known as protein kinase B, is a serine/threonine kinase involved in a wide range of cellular functions including survival, metabolism, and immune responses [27]. In the context of inflammatory bowel diseases, AKT signaling modulates the polarization of macrophages, a key process in determining inflammatory versus reparative immune phenotypes. Specifically, AKT1 activation has been associated with anti-inflammatory M2 polarization, whereas AKT2 promotes proinflammatory M1 polarization and the production of cytokines such as IL-1β and TNF-α [28]. Dysregulation of AKT phosphorylation can thus exacerbate mucosal damage and immune imbalance. However, its role in the pathophysiology of HAEC remains largely unexplored.

This study aims to investigate the role of MSC in HAEC and its relationship with macrophage, providing experimental foundations for the prevention and treatment of HAEC.

## 2. Materials and Methods

### 2.1. Human Samples

After obtaining ethics approval from the Ethics Committee of Children's Hospital, Zhejiang University School of Medicine and written informed consent from the patient's parents, intestinal samples from eight patients with HSCR were collected during surgery between November 2022 and June 2023 in the Department of Neonatal Surgery, Children's Hospital, Zhejiang University School of Medicine. The gravity of enterocolitis was assessed using the histopathological grading system established by Elhalaby et al. [29] which correlates clinical severity with microscopic findings. Subsequent to evaluate the degree of inflammation through histopathological examinations, specimens were segregated into a HSCR group and a HAEC group [29] (Figure S1).

### 2.2. Murine Model of HSCR

The breeding cohort of heterozygous mice (Ednrb^tm1Ywa/J^, with a 6J-129Sv background) was procured from Professor JieXiong Feng at Tongji Hospital, Tongji Medical College, Huazhong University of Science and Technology. Genetic identification of the mice was conducted through PCR15. The primers used were as follows: Mutant-specific primer: 5′-ATAGATTCGCCCTTGTGTCC-3′, Wild-type-specific primer: 5′-GATGAACCTGTGCTCAGTGCAA-3′, Common primer: 5′-CATGGTC TTGTTTCCTGATGC-3′. The PCR reaction system included 10 μL of 2× PCRMix, 0.5 μL each of the mutant, wild-type, and common primers, 1.5 μL of template DNA, and 7 μL of ddH_2_O, for a total volume of 20 μL. The thermal cycling conditions were as follows: 94°C for 5 min (initial denaturation), followed by 35 cycles of 94°C for 30 s, 56°C for 30 s, and 72°C for 30 s, with a final extension at 72°C for 10 min, and a hold at 4°C. PCR products were stored at −20°C.

Heterozygous mice (Ednrb^+/−^) served as the breeding stock, while wild-type (WT, Ednrb^+/+^) and homozygous (Ednrb^−/−^) mice were designated as experimental subjects. At around 3–4 weeks after birth, Ednrb^−/−^ mice might succumb to severe HAEC. Therefore, when mice manifest at least two of the following symptoms: perspiration, bloody stools, reduced body temperature, diminished activity and food intake, and lack of weight gain for two consecutive days, they were promptly euthanized, and their colonic tissues were dissected for subsequent experiments. The enterocolitis grading system was employed to assess the severity of colitis [15, 30].

### 2.3. Mouse Treatment

On the 18^th^, 20^th^, and 22^nd^ days following birth, enemas of either PBS or MSC-derived exosomes were administered to Ednrb^−/−^ mice twice daily, with each dose consisting of 100 μL. The exosomes, diluted in PBS, were infused at a concentration of 2 mg/mL [31]. Following this regimen, the survival times of a subset of mice were meticulously documented posthumously, while another subset was euthanized at 28 days of age, with their colonic tissues and serum meticulously harvested for subsequent experimentation.

### 2.4. Hematoxylin and Eosin (HE) Staining

HE staining was carried out to assess the degree of intestinal lesions. All obtained colonic samples were fixed with 4% paraformaldehyde for 24–48 h, sliced after paraffin embedding, stained with HE, and examined using an Olympus light microscope (Olympus, Hachiko, Japan). Histological changes in the terminal ileum were used to diagnose.

### 2.5. Immunohistochemical Staining

Immunohistochemical staining was performed as previously described [32]. The paraffin sections were incubated overnight at 4°C with primary antibodies, anti-CD68 (Santa Cruz, USA), followed by incubation with horseradish peroxidase-coupled secondary antibodies at room temperature for 1 h. Finally, DAB solution (Sangon Biotech, Shanghai, China) and hematoxylin were used for staining. High-resolution digital images were captured with optical microscope (Olympus, Hachiko, Japan).

### 2.6. Immunofluorescence Staining

After the tissues were blocked with 5% BSA for 1 h, they were incubated overnight with anti-CD68 and anti-p-AKT (CST, Boston City, USA) followed by staining with Alexa Fluor 488-labeled and 594-labeled secondary antibodies (Jackson Immunoresearch, PA, USA). The nuclei were counterstained with 4,6-diamidino-2-phenylindole (BBI, Shanghai, China) and then viewed under a Zeiss microscope (Carl Zeiss, Jena, Germany).

### 2.7. Isolation of Mouse Intestinal Macrophages

Intestinal leukocytes of the Ednrb^−/−^ and WT mice, were isolated as described previously [33]. Single-cell suspensions were incubated with combinations of antibodies fixable viability stain F4/80, CD11b, and CD45 antibodies (Invitrogen, Carlsbad, USA) for flow cytometry analysis.

### 2.8. Cell Extraction and Culture

Two-week-old SD rats (Shanghai Slac Laboratory Animal Company, Shanghai, China) were sacrificed by spinal dislocation. After separating the femur and tibia, subtracted the epiphysis and flushed the bone marrow cavity to obtain MSCs suspension. MSCs were cultured in DMEM/F12 (1:1) medium containing 10% fetal bovine serum (Ausgenex, Australia) and 1% penicillin/streptomycin (Sangon Biotech, Shanghai, China). The MSCs were maintained in an incubator at 37°C and 5% CO_2_. The culture medium was replaced every 2 days. When the cells were 80% confluent, MSCs were digested with trypsin for passaging, and MSCs in passages 2–6 were used for the experiments. Macrophage (RAW 264.7) and intestinal epithelial cell (IEC-6) lines were obtained from the american type culture collection (ATCC) and Cell Bank in Shanghai, China. Both cells were cultured in DMEM supplemented with 10% fetal bovine serum (Ausgenex, Australia) and 1% penicillin/streptomycin (Sangon Biotech, Shanghai, China).

### 2.9. Isolation of Exosomes and Endocytosis of Exosomes Into Macrophages

Following a previously described protocol [34]. After MSCs reached 80% confluence, exosomes were extracted by washing the cells twice with PBS, followed by re-culturing and incubation for 48 h in the presence of exosome-free serum medium. Following supernatant extraction, exosomes were isolated using differential centrifugation. Centrifugation at 2000 × *g* for 10 min to remove cell debris; 10,000 × *g* for 10 min to remove apoptotic bodies, and 100,000 × *g* for 90 min to remove the supernatant and collect impurities-containing exosomes. The collected exosomes were then washed twice by suspending them in 20 mL PBS and centrifuging them again at 100,000 × *g* for 90 min. Exosomes were then suspended in 100 mL PBS and stored at −80°C until further use.

The size distribution of exosomes were determined using a Nano Sight NS500 (Malvern), and exosomal morphology was examined by employing transmission electron microscopy (Thermo Scientific Talos L120C). To determine whether RAW264.7 cells may endocytose exosomes, exosomes were labeled with PKH67 (PKH67 Green Fluorescent Cell Linker Mini Kit) and then co-cultured with RAW264.7 cells for 24 h. After washing with PBS, RAW264.7 cells were stained for 30 min with DAPI. A fluorescence microscope was used to view and photograph the result (Zeiss, Germany).

### 2.10. Cell Transfection

The miR-mimic-223 or miR-mimic-NC were transfected into RAW264.7 cells with the concentration of 50 nM. And miR-inhibitor-223 or miR-inhibitor-NC were transfected into MSCs. MiRNA was transferred into cells by transfection reagent (Ribo Bio, C10511-05).

### 2.11. RT-qPCR

The extraction of total RNA was performed following our previously published protocol [35]. The quantification of miR-223 was was performed following user manual for bulge-loop miRNA qRT-PCR Primer (RiboBio, RN: R10031.9). The bulge-loop miRNA RT-PCR primer sets were designed by RiboBio. U6 was utilized as an internal control for miRNAs.

### 2.12. Preparation of Macrophage Supernatants

RAW 264.7 cells were seeded at 5 × 10^5^ cells/mL in 6-well plates or 2 × 10^5^ cells/mL in 12-well plates, and IEC-6 cells were seeded at 1 × 10^5^ cells/mL in 12-well plates. After 24 h, RAW 264.7 cells were treated with or without 1 µg/mL LPS (LPS; L2654, Sigma‒Aldrich, MO, USA). After 24 h, the supernatants of macrophages collected were labeled L-MS and MS. In the co-culture system, stem cells were seeded in transwell with 5 × 10^4^/mL, and macrophages were seeded at the bottom of 12-well plate at 2 × 10^5^/mL. After 24 h, the co-culture system was treated with or without 1 µg/mL LPS for 24 h. And the supernatants were labeled L + MSC-MS and MSC-MS. In order to inhibit the secretion of stem cell exosomes [36, 37], 10 μM GW4869 was applied in MSC culture for 24 h before co-culture with macrophages. The remaining steps were the same as above. And the supernatants were labeled L + MSC + GW4869-MS and MSC + GW4869-MS. To verify the effect of exosome, macrophages were pretreated with exosomes of 400 μg/mL concentration for 24 h and then stimulated with or without LPS [31]. The supernatants were labeled L + EXO-MS and EXO-MS. MK2206(100 nM) was add into the RAW 264.7 cells with or without LPS for 24 h, the supernatants were labeled L + MK2206-MS and MK2206-MS [38]. 24 h after cell transfection, macrophages were stimulated with 1 μg/mL LPS, and the collected supernatants were labeled L + miR-mimic-223-MS and L + miR-mimic-NC-MS; MSCs continue to be cultured and exosomes were extracted to treat macrophages. The macrophages were then stimulated with 1 μg/mL LPS, and the obtained supernatants were labeled L + EXO-miR-inhibitor-223-MS and L + EXO-miR-inhibitor-NC-MS. All the supernatants were collected by centrifugation at 7300 g for 15 min and used in subsequent experiments. All the treated cells were collected for subsequent analysis.

### 2.13. Flow Cytometry

Treated macrophages and intestinal epithelial cells were double-stained with annexin V-fluorescein isothiocyanate and propidium iodide (566547, FITC Apoptosis Detection Kit I; BD, CA, USA) to assess cell death. Quantification was then performed using a FACS Lyric flow cytometer (663518, BD, NJ, USA) and analyzed using Flow Jo V10 software.

### 2.14. Western Blot

Total protein was extracted from macrophages with different treatments or tissues using radioimmunoprecipitation assay lysis buffer (Boster, Wuhan, China), and the concentration of proteins was determined by a BCA protein assay kit (Meilunbio, Dalian, China). Cell lysates were resolved by SDS‒PAGE, and polyvinylidene difluoride (Immobilon-P, Merck, USA) membranes were probed with anti-akt, anti-p-akt, anti-mTOR, or anti-p-mTOR antibodies, and then developed by chemiluminescence (Thermo Fisher Scientific, MA, USA). Relative protein levels were quantified with the Gene Sys Chemi imaging system (Syngene G; BOX, USA) and ImageJ (National Institutes of Health, MD, USA).

### 2.15. Enzyme-Linked Immunosorbent Assay (ELISA)

The amount of IL-1β protein in the culture supernatants was measured using mouse-specific IL-1β ELISA kits (abs520001, Absin, Shanghai, China) according to the manufacturer's instructions.

### 2.16. Statistical Analysis

All experiments were performed independently at least three times. GraphPad Software was applied to analyze data by two-tailed unpaired Student's *t*-tests or one-way ANOVA tests by Brown–Forsythe test. Data are presented as the mean ± SEM. *⁣*^*∗*^*p* < 0.05, *⁣*^*∗∗*^*p* < 0.01, *⁣*^*∗∗∗*^*p* < 0.001, *⁣*^*∗∗∗∗*^*p* < 0.0001, SEM standard error of the mean, and NS no significant difference.

## 3. Results

### 3.1. Macrophages Accumulated in HAEC Colon and Were Required for Enterocyte Death

**Figure 1:**
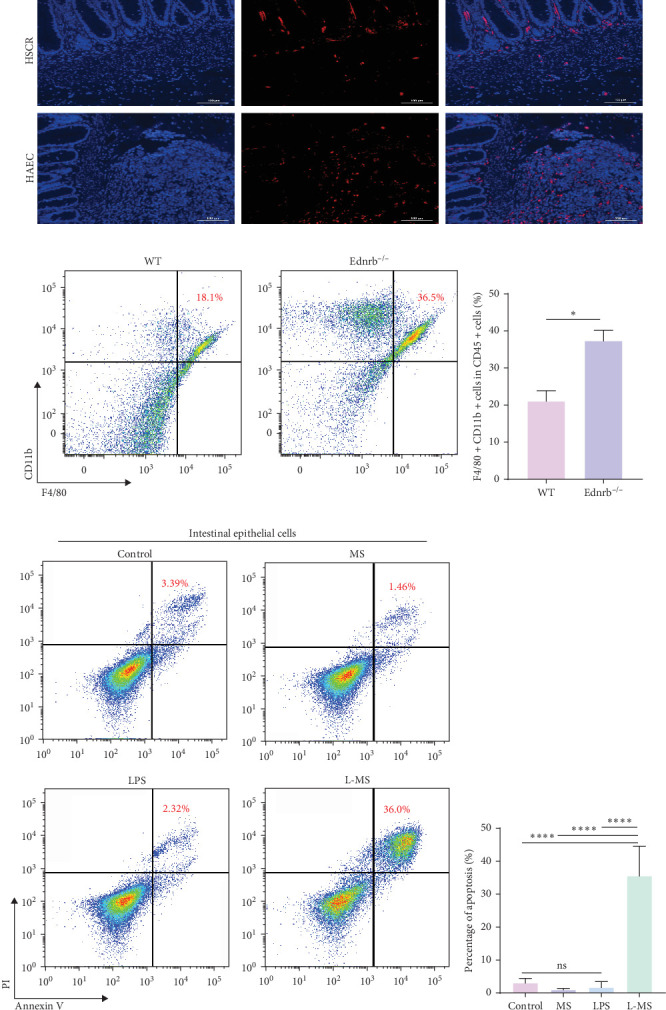
Macrophages accumulated in intestine of HAEC and were required for the death of intestinal epithelial cells. (A) Immunofluorescence staining of colonic tissues from HSCR (control) and HAEC patients. Blue represents nuclei (DAPI), and red represents CD68^+^ macrophages. HSCR tissues were used as the control for comparison to assess macrophage accumulation specific to HAEC. (B, C) Flow cytometry analysis of F4/80^+^CD11b^+^ macrophages within CD45^+^ leukocytes in colonic tissues of wild-type (WT, control) and HAEC mice. WT mice served as controls to determine the pathological increase of macrophages in HAEC. (D, E) Intestinal epithelial cells were stimulated for 24 h with control medium, macrophage supernatant (MS), lipopolysaccharide (LPS), or LPS-stimulated macrophage supernatant (L-MS). Apoptosis was analyzed by flow cytometry. Among these, the unstimulated control group (control medium) was the primary negative control, MS and LPS served as partial controls to distinguish the individual contributions of macrophage-derived factors or LPS alone, while L-MS represented the full experimental condition. For more group information, please refer to the methods section “Preparation of macrophage supernatants.” Data are presented as mean ± SEM. *⁣*^*∗*^*p* < 0.05, *⁣*^*∗∗*^*p* < 0.01, *⁣*^*∗∗∗*^*p* < 0.001, and ⁣^*∗∗∗∗*^*p* < 0.0001; NS, not significant.

Immunofluorescence analysis exhibited a notable augmentation of the macrophage marker CD68 in the colonic tissues of HAEC patients (Figure 1A). Throughout the progression of HAEC in Ednrb^−/−^ mice, there was an increased proportion of F4/80^+^CD11b^+^ cells within the CD45^+^ cell population in the colon (Figure 1B, C), implying the participation of macrophages in the initiation and advancement of HAEC. In vitro assays revealed that the supernatant from LPS-stimulated macrophages induces intestinal epithelial cell apoptosis (Figure 1D, E; L-MS), while the supernatant from normally cultured macrophages (Figure 1D, E; MS) or direct LPS stimulation (Figure 1D, E; LPS) failed to provoke such response, underscoring the pivotal role of macrophages in enterocyte death and mucosal barrier impairment.

### 3.2. MSCs Alleviated Macrophage-Induced Enterocyte Death Through Exosomes

**Figure 2:**
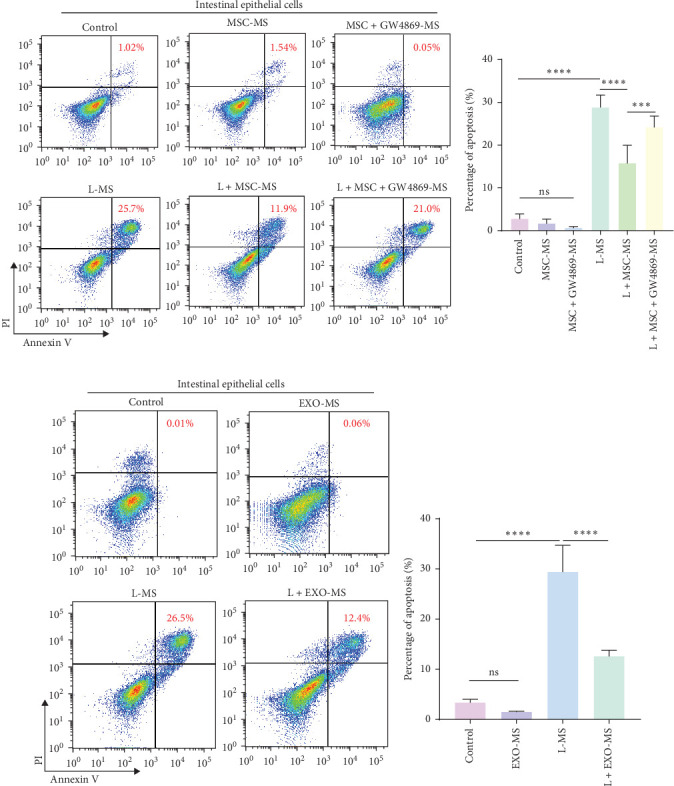
Mesenchymal stem cells attenuated macrophages induced intestinal epithelial apoptosis via exosomes. (A, B) After coculture with stem cells, macrophage-induced enterocyte death decreased, whereas the exosome inhibitor GW4869 could suppress the effects of stem cells. Flow cytometry depicts the proportion of enterocyte death after 24 h of stimulation with supernatants from each group of macrophages. (C, D) MSC-derived exosomes counteract macrophage-induced enterocyte death. Flow cytometry illustrates the status of enterocyte death after 24 h of stimulation with supernatants from each group of macrophages. Control in the Figures means that intestinal epithelial cells were stimulated for 24 h with supernatants collected from unstimulated macrophages. For more group information, please refer to the methods section. Data are presented as the mean ± SEM. *⁣*^*∗*^*p* < 0.05, *⁣*^*∗∗*^*p* < 0.01, *⁣*^*∗∗∗*^*p* < 0.001, *⁣*^*∗∗∗∗*^*p* < 0.0001, NS, no significant difference; SEM, standard error of the mean.

Upon conditioning by MSCs, macrophages exhibited a notable decrease in the induction of enterocyte death (Figure 2A, B), indicating the stem cells' capacity to alleviate epithelial cell demise through modulation of macrophage functionality. Conversely, upon inhibition of MSC-derived exosomal secretion, the conditioning effect of MSCs waned, resulting in an escalation of macrophage-induced enterocyte death (Figure 2A, B), emphasizing the involvement of exosomes in the process of MSC-mediated inhibition of epithelial cell death. To further corroborate the role of MSC-derived exosomes, co-culturing of isolated MSC-derived exosomes with macrophages demonstrated a reduction in the proportion of macrophage-induced enterocyte death (Figure 2C, D), highlighting the direct inhibitory impact of exosomes on macrophage-induced epithelial cell damage.

### 3.3. Exosomes miR-223 Inhibited Macrophage-Induced Enterocyte Death

**Figure 3:**
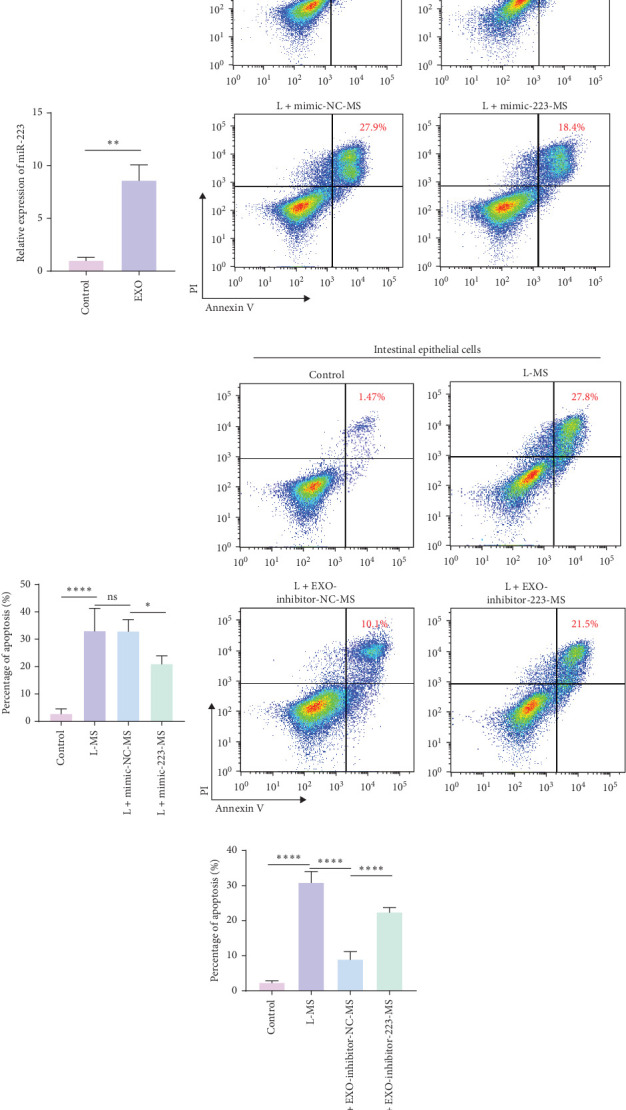
The miR-223 encapsulated within MSC-derived exosomes exerts inhibitory effects on macrophage-induced apoptosis of intestinal epithelial cells. Flow cytometry data illustrate the survival status of intestinal epithelial cells following 24 h of exposure to various macrophage supernatants. (A) The expression of miR-223 in macrophages posttreatment with MSC-derived exosomes was assessed using q-PCR. Control means unstimulated macrophages. (B, C) Following miR-223 transfection into macrophages, the proportion of enterocyte death induced by macrophages was determined. (D, E) Upon inhibition of miR-223 within MSC-derived exosomes, the percentage of enterocyte death induced by macrophages was evaluated. For more group information, please refer to the methods section. The “control” in the (B, C, D, and E) diagram refers to that intestinal epithelial cells were stimulated for 24 h with supernatants collected from unstimulated macrophages. Data are presented as the mean ± SEM. *⁣*^*∗*^*p* < 0.05, *⁣*^*∗∗*^*p* < 0.01, *⁣*^*∗∗∗*^*p* < 0.001, *⁣*^*∗∗∗∗*^*p* < 0.0001, NS, no significant difference; SEM, standard error of the mean.

During the modulation of macrophage immune responses by MSC-derived exosomes, miR-223 is transported into macrophages, promoting their polarization towards an anti-inflammatory M2 phenotype [39]. Cultivating MSC-derived exosomes at a concentration of 400 μg/mL with macrophages for 24 h notably augmented the intracellular abundance of miR-223 in macrophages (Figure 3A). To further affirm the significance of miR-223, miR-mimic-223 was employed to induce miR-223 overexpression in macrophages, while miR-inhibitor-223 was used to impede miR-223 function in stem cell exosomes. Supernatants were prepared according to the prescribed protocol. After miR-223 overexpression, there was a reduction in macrophage-induced enterocyte death (Figure 3B, C), whereas inhibition of miR-223 mitigated the impact of MSC-derived exosomes on enterocytes (Figure 3D, E). This implied that MSC-derived exosomes act on macrophages through miR-223 mediation, thereby, alleviating the rate of enterocyte death.

### 3.4. Intracellular AKT Phosphorylation in Macrophages Is Involved in HAEC Inflammation

**Figure 4:**
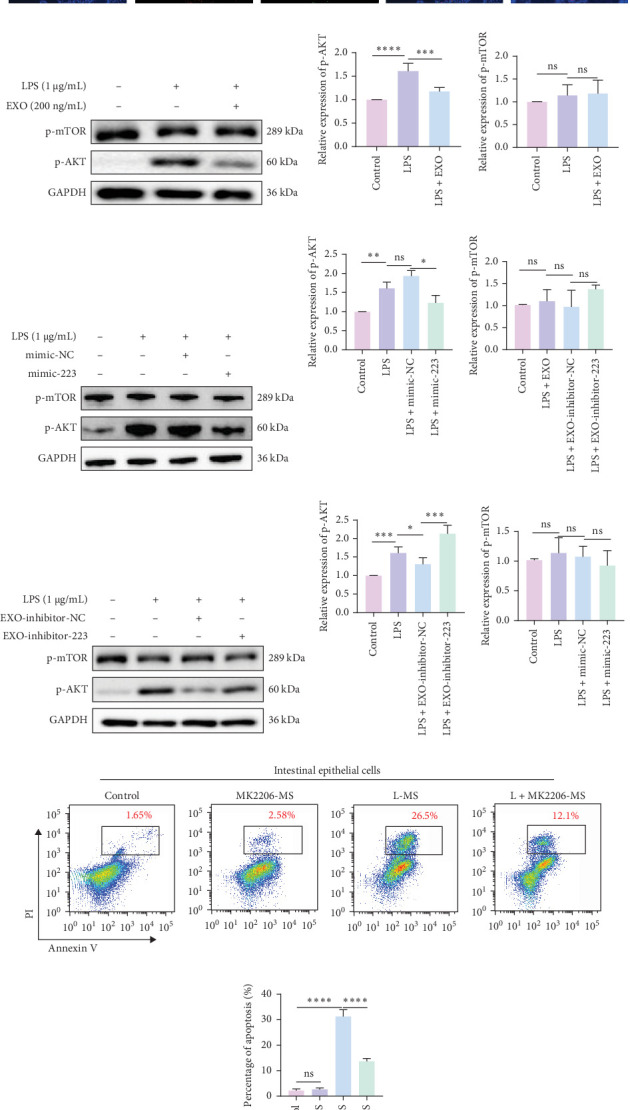
The miR-223 contained within exosomes orchestrates a reduction in macrophage-induced enterocyte death by inhibiting p-AKT signaling. (A) Immunofluorescence staining of colonic tissues from HSCR (control) and HAEC patients. In the setting of HAEC, colonic tissues showcase a marked elevation in p-AKT expression within macrophages. Immunofluorescent staining highlights CD68 in red fluorescence, while p-AKT is depicted in green fluorescence. (B–G) Exosomes, along with their cargo of miR-223, demonstrate the capacity to attenuate p-AKT expression in macrophages. The western blotting results depict the effect of LPS stimulation on macrophages after 24 h. (H, I) The AKT inhibitor MK2206 effectively curtails macrophage-induced enterocyte death. Flow cytometry profiles the survival status of intestinal epithelial cells following 24 h of exposure to various macrophage supernatants. Quantification of western blot band intensities was performed using densitometry, and the results were expressed as relative expression (normalized to GAPDH). Data are presented as the mean ± SEM. *⁣*^*∗*^*p* < 0.05, *⁣*^*∗∗*^*p* < 0.01, *⁣*^*∗∗∗*^*p* < 0.001, *⁣*^*∗∗∗∗*^*p* < 0.0001, NS, no significant difference; SEM, standard error of the mean.

The phosphorylation of AKT played a pivotal role in the inflammatory process, as evidenced by previous studies demonstrating that miR-223 modulated AKT phosphorylation through targeting the CBLB, PTEN, and EGFR pathways, thereby alleviating inflammation [40–42]. This study unveiled that in colonic tissues of HAEC mice, CD68 expression was elevated, with a concomitant increase in p-AKT signal intensity. Immunofluorescence co-staining revealed clear colocalization of CD68 and p-AKT (Figure 4A), indicating the involvement of AKT phosphorylation within macrophages in the inflammatory progression of HAEC. Upon LPS stimulation, there was a notable upsurge in p-AKT expression in macrophages (Figure 4B, C; *p* < 0.0001). However, when LPS-stimulated macrophages were treated with MSC-derived exosomes, p-AKT expression was significantly reduced (*p*=0.0001). Moreover, p-AKT expression was significantly reduced in LPS-stimulated macrophages transfected with miR-223 mimic compared to those transfected with negative control mimic (Figure 4D, E; *p*=0.0326). Conversely, inhibition of miR-223 in MSC-derived exosomes resulted in elevated p-AKT expression in LPS-treated macrophages, compared to exosomes transfected with negative control inhibitor (Figure 4F, G; *p*=0.0003). The modulation of p-AKT expression did not elicit alterations in downstream protein p-mTOR expression, implying that LPS stimulation did not trigger the activation of the AKT/mTOR-associated anti-inflammatory pathway in macrophages. To substantiate the involvement of AKT phosphorylation in macrophage-mediated enterocyte death, the inhibition of AKT within macrophages using MK2206 resulted in a notable decrease in the percentage of induced enterocyte death (Figure 4H, I).

### 3.5. MSCs Derived Exosomes Alleviate HAEC by Reducing IL-1β

**Figure 5:**
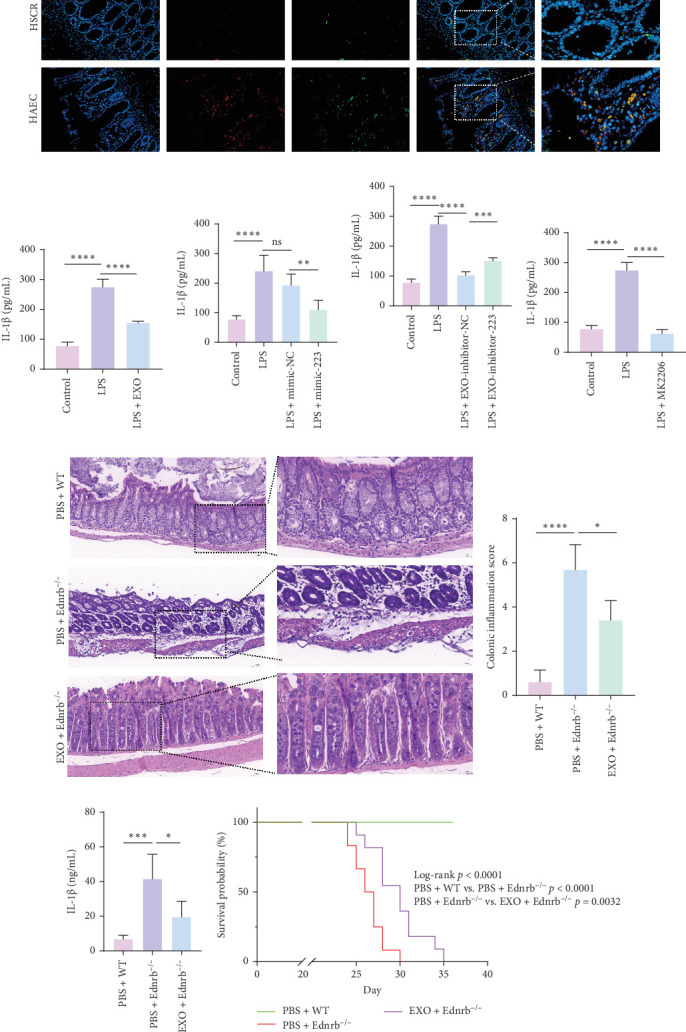
Exosomes derived from mesenchymal stem cells reduce the level of IL-1β in macrophage supernatant and serum of Ednrb^−/−^ mice. (A) Immunofluorescence staining of colonic tissues, where blue represents cell nuclei, red represents CD68, and green represents IL-1β. Images 1 and 2 represent magnified views of the merged fluorescence channels. (B, C, E) Respectively, denote the concentration of IL-1β in the supernatant after treating macrophages with exosomes, miR-223, and MK2206, followed by 24 h of LPS stimulation. (D) The concentration of IL-1β in the supernatant after treating macrophages with exosomes where miR-223 has been inhibited, followed by 24 h of LPS stimulation. (F, G) Exosomes ameliorate colonic inflammation scores in HAEC model mice. Hematoxylin and eosin staining images depict mouse colonic tissues under low and high magnification. (H) Exosomes reduce the level of IL-1β in serum of EDNRB^−/−^ mice. (I) Survival curve of HAEC model mice following enema administration of MSC drived extracellular vesicles. Data are presented as the mean ± SEM. *⁣*^*∗*^*p* < 0.05, *⁣*^*∗∗*^*p* < 0.01, *⁣*^*∗∗∗*^*p* < 0.001, *⁣*^*∗∗∗∗*^*p* < 0.0001, NS, no significant difference; SEM, standard error of the mean.

Our preliminary investigations have unveiled that in the cascade of events where macrophage activation instigates enterocyte death, the inflammatory cytokine IL-1β, exuded by macrophages, assumes a pivotal role [43]. This study also found a notable elevation in IL-1β levels in the colonic tissues of HAEC patients (*n* = 4 per group; Figure 5A). In our in vitro experiments, ELISA assays revealed a marked increase in IL-1β levels in the supernatant of macrophages following LPS stimulation. Nevertheless, interventions involving exosomes, miR-223, or the AKT inhibitor MK2206 induce a notable reduction in IL-1β concentration within the macrophages' supernatant (Figure 5B, C, E). Conversely, inhibition of miR-223 within exosomes prompted a perceptible escalation in IL-1β concentration (Figure 5D). In the vivo experiments, the serum IL-1β concentration in Ednrb^−/−^ mice was significantly elevated, whereas following exosomes administration, there was a reduction in serum IL-1β levels in the mice (*n* = 5 per group; Figure 5H). The colonic tissues of Ednrb^−/−^ mice at 4 weeks exhibited severe inflammatory cell infiltration, which showed disrupted colonic mucosal architecture, extensive inflammatory cell infiltration, ulceration, and even perforation, with inflammation involving the muscular and serosal layers (Figure 5F, G). In contrast, the exosomes enema group showed restoration of mucosal architecture and reduced inflammatory cell infiltration. Exosomes infusion significantly lowered the intestinal inflammation score in Ednrb^−/−^ mice (Figure 5F, G). In terms of survival curves, the median survival days for the PBS + Ednrb^−/−^ group (*n* = 12) was 26.5 days, whereas the EXO + Ednrb^−/−^ group (*n* = 11) was 30 days (Figure 5I). Exosome enema delayed the death of HAEC mice to some extent.

## 4. Discussion

HAEC is a severe and potentially life-threatening complication of Hirschsprung disease, characterized by bowel inflammation, epithelial damage, and immune dysregulation [44]. Clinically, HAEC manifests with abdominal distension, fever, and diarrhea, and is a leading cause of morbidity and mortality in HSCR patients. Despite surgical correction of aganglionosis, HAEC frequently recurs [45], indicating that postoperative pathophysiological processes, particularly involving the intestinal immune microenvironment, play a central role in its onset and progression. Recent studies have emphasized the importance of immune cell infiltration in the pathogenesis of HAEC [15, 46, 47].

This investigation reveals a conspicuous aggregation of macrophages in the colon of HAEC-afflicted children, concomitant with an upregulation of AKT phosphorylation. Macrophages stimulated by LPS drive epithelial cell death during the onset of enteritis. Within the process of macrophage-induced epithelial cell death, miR-223 within MSC-derived exosomes orchestrates a downregulation of macrophage AKT phosphorylation, thereby, inhibiting the secretion of IL-1β and diminishing the proportion of enterocyte death. In vivo experiments substantiate that MSC-derived exosomes attenuate the proportion of macrophages in the colonic tissues of HAEC mice, thereby, ameliorating intestinal inflammation. Notably, HAEC mice exhibit no overt immunological rejection following repeated administration of rat-derived exosome enemas, thereby, furnishing robust experimental evidence for the clinical application safety of MSC-derived exosomes.

Throughout the course of enteritis, a multitude of immune cells undergo alterations. During the onset of inflammatory bowel disease, neutrophils are recruited to inflammatory sites by chemotactic factors released by intestinal epithelial cells, unleashing reactive oxygen species and neutrophil elastase, thereby, disrupting cell membranes and intercellular connections, culminating in crypt distortion, and abscess formation [48]. Macrophages play a role akin to neutrophils in the cascade of inflammation, marshaling inflamed cells, and unleashing inflammatory mediators to breach the intestinal epithelial barrier, exacerbating intestinal inflammation [12–16]. However, the occurrence of HAEC appears to be intricately linked to the presence of macrophages within the intestines, where macrophages, polarized towards the M1 phenotype, release inflammatory cytokines damaging intestinal epithelial cells and Cajal cells, playing a pivotal role in triggering and exacerbating the progression of HAEC [15, 49]. This study also confirms the substantial recruitment of macrophages to colonic tissues during HAEC onset.

Presently, research concerning the immunosuppressive role of MSCs posits that only MSCs activated by inflammatory factors exhibit heightened immunosuppressive capabilities [50], while stimulation by LPS activates TLR 4 in MSCs, thereby, abolishing their inhibition of peripheral blood mononuclear cell proliferation [51]. This study reveals that even after exposure to LPS stimulation within the co-culture system of macrophages and MSCs, MSCs retain the ability to suppress the pro-inflammatory response of macrophages. This indicates that the activation of MSCs by inflammatory factors released by LPS stimulation inhibits the inflammatory progression of macrophages themselves [50, 52]. However, there is another plausible scenario, prior to LPS stimulation, MSCs may have already completed the domestication of macrophages [53]. This study employs exosome pretreatment of macrophages with MSCs not exposed to LPS stimulation and intraluminal infusion of exosomes before the onset of HAEC in a murine model of intestinal inflammation, thereby, excluding interference from LPS and inflammatory factor stimulation on MSCs, demonstrating that MSCs and their exosomes in a quiescent state can also domesticate macrophages, exerting an anti-inflammatory effect. This finding provides experimental evidence for the clinical prevention of HAEC occurrence using MSC-derived exosomes.

MSCs exert immunomodulatory effects through their exosomes, primarily composed of miRNA [54]. In models of acute kidney injury, MSC-derived exosomes mitigate inflammation and injury by downregulating the expression of CX3C chemokine receptor 1 through miR-15a, miR-15b, and miR-16, thus, inhibiting TLR signal transduction and macrophage recruitment [54, 55]. This study reveals that within HAEC colonic tissues, there is an upregulation of AKT phosphorylation in macrophages; however, miR-223 within MSC-derived exosomes counteracts this by diminishing AKT phosphorylation in macrophages, suppressing the secretion of IL-1β, and reducing macrophage-induced epithelial cell death.

miR-223 plays a pivotal role in the activation and polarization of macrophages. The majority of studies indicate that it orchestrates macrophage polarization towards the M2 phenotype by selectively targeting the expression of genes such as *Nfat5*, *Rasa1*, *Pknox1*, and *TRAF6*, thus, inhibiting their M1 polarization [39, 56, 57]. However, some research suggests that miR-223, by targeting STAT expression, promotes the secretion of IL-1β and IL-6 induced by LPS, exacerbating tissue inflammation and injury [58, 59]. The experimental findings of this study confirm that post-treatment with miR-223 inhibits the pro-inflammatory response of macrophages, resulting in a reduction in the concentration of IL-1β in the cell supernatant.

miR-223′s modulation of AKT presents varying conclusions across different studies. In epidermal and neuronal cells, miR-223 promotes AKT phosphorylation, thereby inhibiting apoptosis [41]; conversely, in cardiac myocytes, it mitigates myocardial inflammation by downregulating AKT phosphorylation [60]. This experimental evidence demonstrates that miR-223 within MSC-derived exosomes inhibits AKT phosphorylation in macrophages, thus, ameliorating the inflammatory process. The PI3K/AKT pathway and its downstream targets serve as pivotal regulators of macrophage activation phenotype; activated AKT can activate mTOR by inhibiting TSC and suppress macrophage activation through NF-kB pathway inhibition [61]. However, the effects post-activation of different subtypes of PI3K and AKT vary. Notably, activation of AKT1 promotes M2 polarization in macrophages, while AKT2 activation induces a pro-inflammatory response [28, 62, 63]. Experimental findings from this study indicate significant AKT activation in macrophages from HAEC colonic tissues and LPS-stimulated macrophages, with significant inhibition of inflammatory cytokine secretion upon AKT suppression, suggesting AKT2 as the primary activated AKT subtype in this process. Moreover, miR-223 does not directly target AKT but influences its phosphorylation by modulating upstream protein signaling pathways. Across different cells and tissues, miR-223 acts on diverse targets, leading to inconsistent effects on AKT phosphorylation.

The previous investigations conducted by our research team unveiled IL-1β as the primary inflammatory mediator induced by macrophages, leading to epithelial cell death within the intestinal lining [43]. This study corroborates that miR-223, harbored within MSC-derived exosomes, modulates IL-1β secretion by downregulating macrophage AKT phosphorylation. In the context of fungal keratitis, miR-223 targets the autophagy-related gene ATG16L1, dampening the autophagic activity of corneal stromal cells and murine corneal tissues, exacerbating keratitis, thereby elevating IL-1β levels within the tissue [64]. It is conceivable that miR-223 may mitigate IL-1β secretion by inhibiting AKT2 phosphorylation in macrophages, or alternatively, activate the AKT1/mTOR pathway to suppress cellular autophagy and increase IL-1β secretion.

There exists a discernible enrichment of extracellular vesicle miRNA compared to their intracellular counterparts. Intracellularly, an inherent sorting mechanism associated with miRNA sequences dictates their retention within the cell or enrichment within extracellular vesicles [65]. By externally modifying miRNA sequences or inserting target sequences, we can alter the directional sorting of functional miRNA, thereby enhancing their accumulation within extracellular vesicles and augmenting the transfer of the targeted miRNA to recipient cells. This avenue of research provides novel insights into immunotherapy for various diseases [65].

This study sheds light on a novel anti-inflammatory mechanism through which MSC-derived exosomes, enriched with miR-223, mitigate macrophage-induced enterocyte death by modulating AKT signaling. These findings not only expand our understanding of HAEC pathogenesis but also highlight the therapeutic potential of exosome-based strategies in preventing or ameliorating HAEC in clinical settings. The observation that repeated administration of rat-derived exosomes caused no apparent immune rejection further supports their translational viability. Looking forward, the next crucial step involves identifying the precise molecular targets of miR-223 within macrophages and validating the efficacy and safety of exosome-based interventions in large-animal models or preclinical humanized systems. Such advances may pave the way for the development of targeted, cell-free therapies for HAEC prevention and treatment.

## 5. Conclusion

In HAEC, macrophages serve as pivotal mediators of enterocyte death. MSC-derived exosomes alleviate HAEC by inhibiting IL-1β secretion through miR-223-mediated suppression of AKT phosphorylation within macrophages.

## Data Availability

The data that support the findings of this study are available from the corresponding author upon reasonable request.

## Supporting Information

Supporting Information In the supporting pictures, we present H&E staining as well as immunohistochemical staining of colonic tissues from HSCR and HAEC patients, which further illustrate histopathological and immune cell differences between the two groups. We attach pictures of stem cell identification, stem cell exosome identification, and macrophage endocytosis of stem cell exosomes. The specific annotations can be seen in Figures S1 and S2.
