# Supplementary material for: Mesenchymal Stem Cell Derived Exosomes Alleviates Hirschsprung-Associated Enterocolitis by Inhibiting AKT Phosphorylation in Macrophages Through miR-223
Source: Stem Cells Int. 2025 Sep 15;2025:3218993. doi: 10.1155/sci/3218993 (PMC12453914; doi:10.1155/sci/3218993)
Supplement: Supporting Information — In the supporting pictures, we present H&E staining as well as immunohistochemical staining of colonic tissues from HSCR and HAEC patients, which further illustrate histopathological and immune cell differences between the two groups. We attach pictures of stem cell identification, stem cell exosome identification, and macrophage endocytosis of stem cell exosomes. The specific annotations can be seen in Figures S1 and S2. [file 3218993.f1.pptx]

## Slide 1
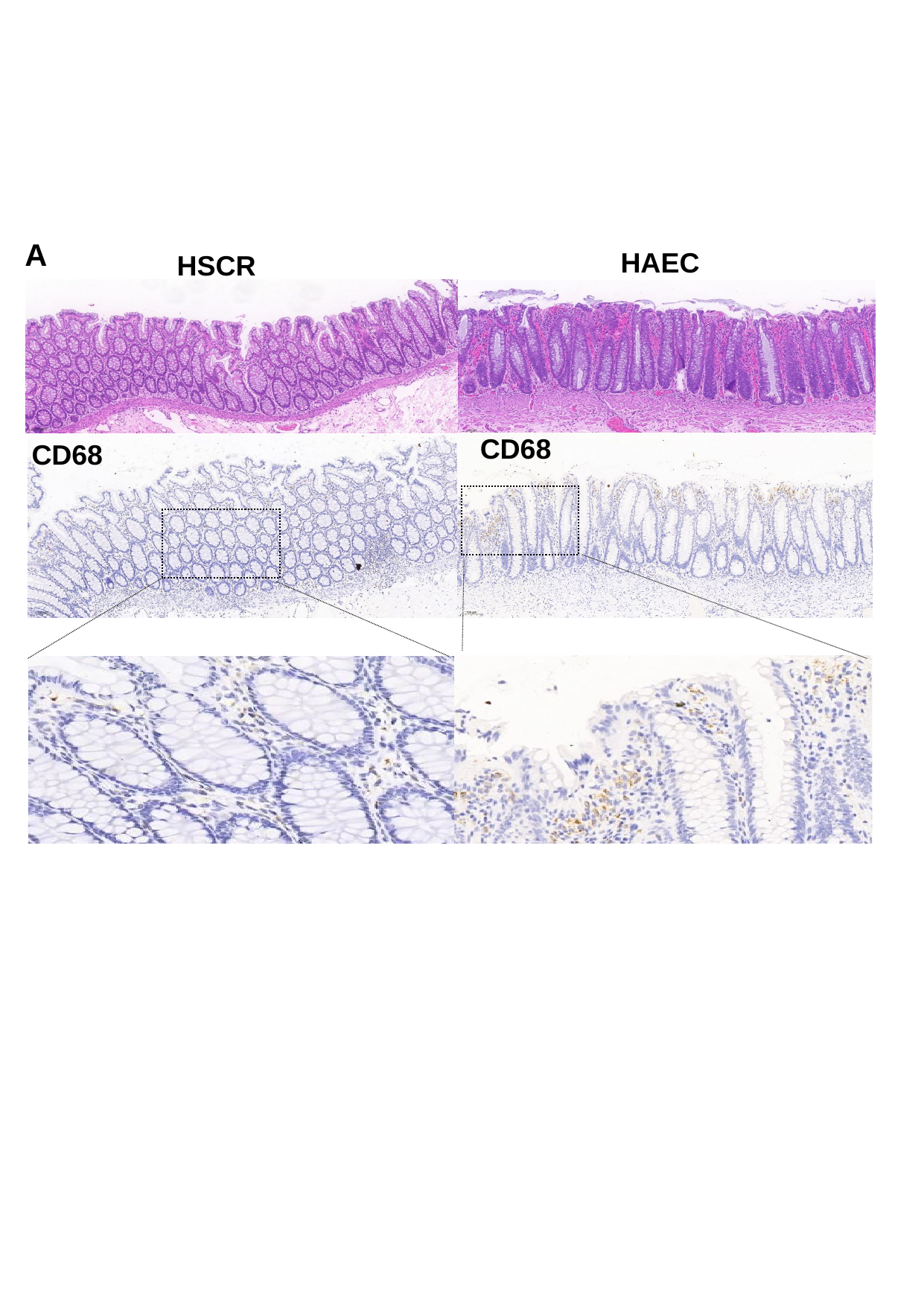

A
HSCR
HAEC
CD68
CD68

## Slide 2
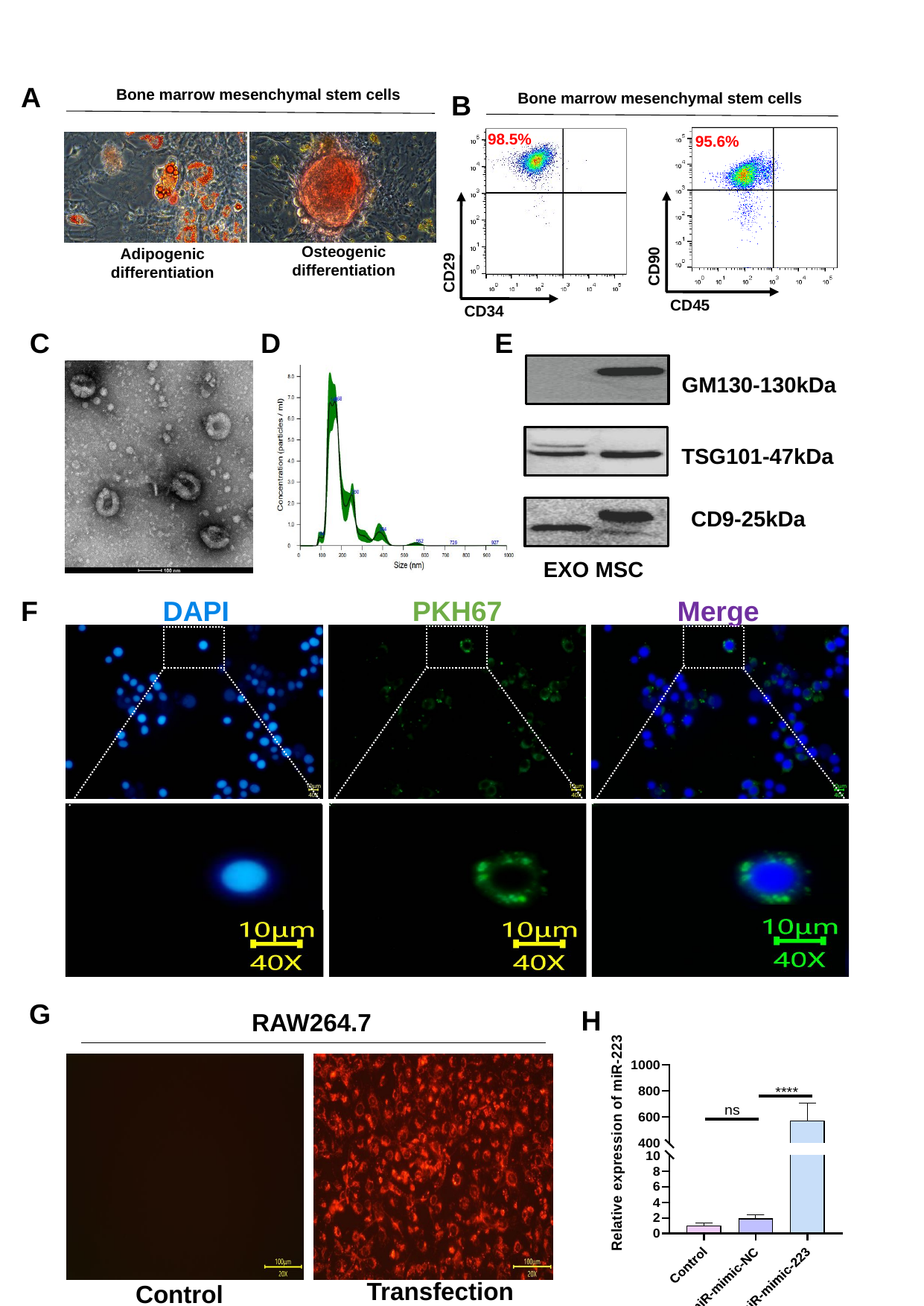

A
Bone marrow mesenchymal stem cells
Osteogenic differentiation
Adipogenic differentiation
Bone marrow mesenchymal stem cells
98.5%
95.6%
CD29
CD34
CD90
CD45
B
C
EXO MSC
GM130-130kDa
TSG101-47kDa
CD9-25kDa
D
E
F
DAPI
PKH67
Merge
G
H
RAW264.7
Transfection
Control

## Slide 3
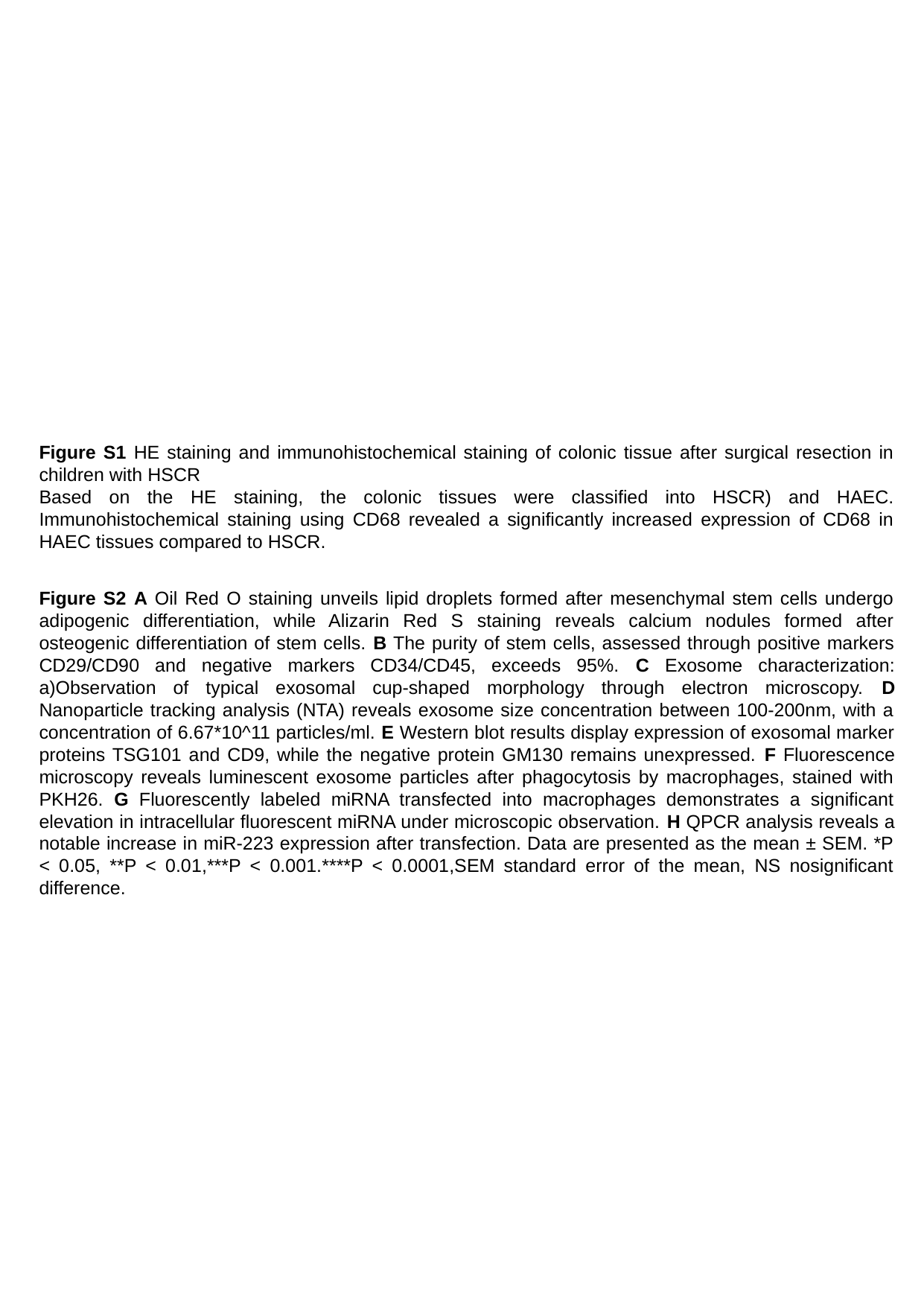

Figure S1 HE staining and immunohistochemical staining of colonic tissue after surgical resection in children with HSCR
Based on the HE staining, the colonic tissues were classified into HSCR) and HAEC. Immunohistochemical staining using CD68 revealed a significantly increased expression of CD68 in HAEC tissues compared to HSCR.
Figure S2 A Oil Red O staining unveils lipid droplets formed after mesenchymal stem cells undergo adipogenic differentiation, while Alizarin Red S staining reveals calcium nodules formed after osteogenic differentiation of stem cells. B The purity of stem cells, assessed through positive markers CD29/CD90 and negative markers CD34/CD45, exceeds 95%. C Exosome characterization: a)Observation of typical exosomal cup-shaped morphology through electron microscopy. D Nanoparticle tracking analysis (NTA) reveals exosome size concentration between 100-200nm, with a concentration of 6.67*10^11 particles/ml. E Western blot results display expression of exosomal marker proteins TSG101 and CD9, while the negative protein GM130 remains unexpressed. F Fluorescence microscopy reveals luminescent exosome particles after phagocytosis by macrophages, stained with PKH26. G Fluorescently labeled miRNA transfected into macrophages demonstrates a significant elevation in intracellular fluorescent miRNA under microscopic observation. H QPCR analysis reveals a notable increase in miR-223 expression after transfection. Data are presented as the mean ± SEM. *P < 0.05, **P < 0.01,***P < 0.001.****P < 0.0001,SEM standard error of the mean, NS nosignificant difference.
